# Cross-Genome Comparisons of Newly Identified Domains in *Mycoplasma gallisepticum* and Domain Architectures with Other *Mycoplasma* species

**DOI:** 10.1155/2011/878973

**Published:** 2011-08-08

**Authors:** Chandra Sekhar Reddy Chilamakuri, Adwait Joshi, Sane Sudha Rani, Bernard Offmann, R. Sowdhamini

**Affiliations:** ^1^Equipe de Bioinformatique, Laboratoire de Biochimie et Génétique Moléculaire, Université de La Réunion, 15 avenue René Cassin, La Réunion, 97715 Saint Denis Messag Cedex 09, France; ^2^National Centre for Biological Sciences, GKVK Campus, Bellary Road, Bangalore 560065, India

## Abstract

Accurate functional annotation of protein sequences is hampered by important factors such as the failure of sequence search methods to identify relationships and the inherent diversity in function of proteins related at low sequence similarities. Earlier, we had employed intermediate sequence search approach to establish new domain relationships in the unassigned regions of gene products at the whole genome level by taking *Mycoplasma gallisepticum* as a specific example and established new domain relationships. In this paper, we report a detailed comparison of the conservation status of the domain and domain architectures of the gene products that bear our newly predicted domains amongst 14 other *Mycoplasma* genomes and reported the probable implications for the organisms. Some of the domain associations, observed in *Mycoplasma* that afflict humans and other non-human primates, are involved in regulation of solute transport and DNA binding suggesting specific modes of host-pathogen interactions.

## 1. Introduction

Progress in DNA sequencing technology has produced the whole genomes of many important organisms including humans. The proper utilization of such sequence information requires understanding of the function of each protein in the database. The ever-increasing gap between the number of sequences deposited in databases and the numbers with accurate functional annotation is a big concern to the scientific community. The goal of functional genomics is to determine the function of proteins predicted from the sequencing projects [1, 2]. To reach this goal, computational approaches can assist in the classification of functional genomics targets. 

Functional and evolutionary relationships can be inferred from sequence comparisons, especially at high sequence identities. The established computational methods to function detection primarily depend on homology matching to genes with known functions by employing programs such as FASTA [3] and BLAST [4]. 

Nevertheless, establishing homology is not straightforward and provides limited coverage. Over the past few years, many new methods have emerged to organize the proteins; some of them are highly automated, and others are curated. Position-specific iterative BLAST (PSI-BLAST) can be used to extend the search to distantly related homologues [5]. Some of the other methods rely on the hierarchical classification of proteins into families such as the superfamilies/families in the PIR-PSD [6] protein groups in ProtoMap [7]. Few other methods organize proteins to families of domains such as Pfam [8] and SMART [9]. Others rely on sequence motifs or conserved regions, such as in PROSITE [10] and PRINTS [11]. Databases like CATH [12], SCOP [13], and FSSP [14] employ structural data to organize proteins in to domains. Others are integrations of various family classifications, such as InterPro [15]. However, each of these databases is useful for particular needs, and most of them rely on high sequence similarity for accurate function annotation transfer, and no classification scheme is by itself adequate for addressing all genomic annotation needs [16]. The Gene Ontology (GO) consortium provides a controlled vocabulary to describe the function of a protein [17]. 

Identification of domains at the sequence level most often relies on the detection of global and local sequence alignments between a given target sequence and domain sequences found in databases such as Pfam [8] and SMART [9]. However, sequence-based methods often fail under low sequence identity conditions. Intermediate sequence approach has been shown to be more effective in enhancing the coverage in homology search and in connecting remotely related proteins of common function [18]. It was shown that about 70% improvement over direct search [18] is possible using this method. Using similar approach in the domain assignment to sequences, earlier, we showed that the domain assignment could be substantially enhanced in the family of genes containing adenylyl cyclases [19]. PURE, this computation-intensive search protocol, was further developed as a web tool [20]. Next, we had implemented our method at the whole genome level by taking smaller genome organism *Mycoplasma gallisepticum *as a specific example [21]. This paper reports the cross-genome comparisons of 14 *Mycoplasma* genomes to study the conservation of domains and domain architectures involving new domain associations identified by us in *Mycoplasma gallisepticum.*

As shown in the earlier paper, PURE approach is effective in establishing remote domain relationships [20, 21] and can be useful when the user fails to assign domains to the sequence by using direct search methods like Pfam [8]. We also showed, by comparing different versions of Pfam databases, that the PURE approach can give a good hint at the domains, which are going to be assigned in the updated Pfam database [19].

*Mycoplasma* constitutes a unique group of bacteria best characterized as lacking peptidoglycan and having one of the smallest genomes of all free-living prokaryotes. Members of this group also represent important pathogens of humans, animals, and plants. Over the last few years, the genomes of many *Mycoplasma* species were sequenced, reinforcing comparative genome studies which permit a better understanding of their metabolism and the relations with their hosts. Phylogenetic analyses indicate that Mycoplasmas have undergone a degenerative evolution from related, low G+C content, Gram-positive eubacteria [22, 23]. Mycoplasmas possess no complete routes for amino acids synthesis and degradation, implying that these monomers must be acquired either from their hosts or from a culture medium, depending upon membrane transporters [24]. Exogenous peptides are an important source of amino acids. Indeed, bacteria have evolved peptide transport systems that also assist in responses to environmental changes, mediating functions such as quorum sensing, sporulation, pheromone transport, and chemotaxis [25].

## 2. Materials and Methods

Complete protein sequences of 14 different *Mycoplasma* genomes were obtained from National Center for Biotechnology Information website [26]. The species we considered for our study were *Mycoplasma gallisepticum* strain R (total number of proteins in the genome 726), *Mycoplasma genitalium* strain G37 (477), *Mycoplasma agalactiae* strain PG2 (742), *Mycoplasma arthritidis* strain 158L3-1 (631), *Mycoplasma capricolum* subsp. *capricolum *(812), *Mycoplasma hyopneumoniae* strain 232 (691), *Mycoplasma hyopneumoniae* strain 7448 (657), *Mycoplasma hyopneumoniae* strain J (657), *Mycoplasma mobile* strain 163K(633), *Mycoplasma mycoides *subsp*. mycoides* SC str. PG1 (1016), *Mycoplasma penetrans* strain HF-2 (1037), *Mycoplasma pneumoniae* strain M129 (689), *Mycoplasma pulmonis* strain UAB CTIP (782), and *Mycoplasma synoviae *(659) (Table 1). 

*Mycoplasma* species can be categorized into different groups based on motility and host specificity [27]*. Mycoplasma gallisepticum*,* Mycoplasma genitalium*,* Mycoplasma mobile*,* Mycoplasma pneumonia, *and* Mycoplasma pulmonis* were grouped as motile and the remaining species *Mycoplasma agalactiae *PG2,* Mycoplasma arthritidis *158L31,* Mycoplasma capricolum *ATCC 27343,* Mycoplasma hyopneumoniae *232,* Mycoplasma hyopneumoniae *7448,* Mycoplasma hyopneumoniae, Mycoplasma mycoides, Mycoplasma penetrans, *and* Mycoplasma synoviae *53 were grouped as nonmotile. Mycoplasmas were also classified based on the host specificity. *Mycoplasma genitalium, Mycoplasma penetrans, Mycoplasma pneumonia, *and* Mycoplasma pulmonis* were primate specific, *Mycoplasma synoviae_53 *and* Mycoplasma gallisepticum *grouped as avian specific, *Mycoplasma hyopneumoniae *232, *Mycoplasma hyopneumoniae *7448, and* Mycoplasma hyopneumoniae *–J were grouped as swine-specific Mycoplasmas. *Mycoplasma arthritidis 158L3 1 *and* Mycoplasma pulmonis* are grouped as rodent specific, *Mycoplasma agalactiae PG2, Mycoplasma capricolum ATCC 27343, *and *Mycoplasma mycoides* are grouped as ovine specific, and lastly *Mycoplasma mobile *is fish-specific *Mycoplasma* in targeting its host for survival. 

We assigned domain region to the *Mycoplasma gallisepticum* protein sequences by scanning the sequences against HMM profiles in the PfamA database (version 21.0) [8] which consists of 8957 families by using standalone version of Hmmpfam of the HMMER suite [28] with *E*-value cutoff 0.1. 

HMMTOP [29] server was used for transmembrane helix prediction, and a standalone version of COILS [30] program was used for coiled-coil region prediction. We used PSI-BLAST [5] (with three iterations and expectation cut-off value of 0.001) for search for similar sequences. During the blast searches, low complexity filter was turned on. Nonredundant database [31] was used for sequence similarity searches. Standalone version of PSIPRED [32] was used for secondary structure prediction. Multiple sequence alignments were performed using CLUSTALW program [33].

## 3. Results and Discussion

Earlier analysis revealed 71 new domain relationships in the *Mycoplasma gallisepticum* genome which corresponds to 62 unassigned regions [21]. 22 domains, which are in the border regions of cut-off expectation value, were excluded from the cross-genome analysis, and 49 domains which belong to 42 unassigned regions are used in the analysis. Detailed domain architectures, along with newly predicted domains, are shown in Table 2. 24 sequences out of 42 sequences picked up one or more domains, which were initially full-length unassigned sequences. Interestingly, some of the newly predicted domains such as Chase 3, DUF 1393, DUF 30, DUF 31, LMP, and HTH 12 are not present in the other *Mycoplasma* genomes. These domains could only be identified in *Mycoplasma gallisepticum *genome in the indirect searches. This could be because these domains may have species-specific functions or Mollicutes may have evolved by degenerative or reductive evolution, accompanied by significant losses of genomic sequences [34], wherein some of these domains might have lost their function and diverged beyond recognition by direct search methods. The intermediate sequences through which these domain relationships are established are predominantly of prokaryotic in origin and have relatively fewer hits in the PSI-BLAST search.

Our analysis also revealed the presence of extra copy of domains such as RMMBL, Lactamase_B, ABC_membrane, ABC_tran, Lipoprotein_X, SBP_bac_5, ATP_synt_ab_N, Helicase_C, tRNA_anti, and GTP_EFTU in the *Mycoplasma gallisepticum *genome. Because of the limited coding capacity of their genome, Mycoplasmas lack many enzymatic pathways characteristic of most bacteria; consequentially, *Mycoplasma* genes encode many proteins with functions related to catabolism and metabolite transport while encoding few anabolic proteins [35]. Most of these newly predicted domains related to transportation function. Despite low sequence identities, these domains could have critical function in the nutrient transportation. Some of the interesting examples are explained below.

**Figure 1:**
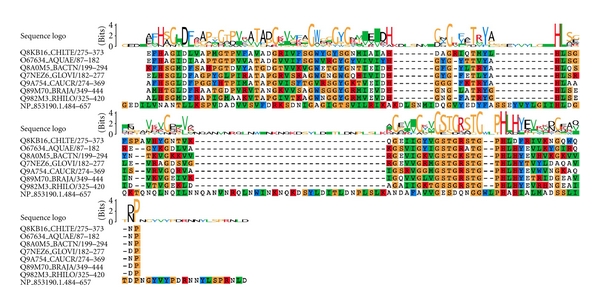
Multiple sequence alignment between peptidase_M23 family representative sequences and unassigned protein sequence (NP_853190.1) from *M. gallisepticum *genome. Peptidase_M23 sequences obtained from Pfam database. Characteristic HxH motif is conserved and has few insertion regions in the unassigned sequence.

**Figure 2:**
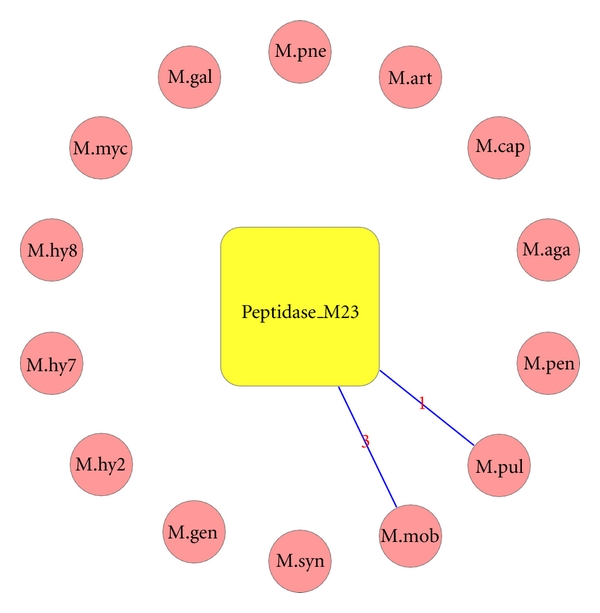
Peptidase domain presence in different *Mycoplasma* genomes. Domain represented by square box and species by circle. Lines connecting domain and species indicate the presence of domain in that species. Edge numbers indicate number of domains copies in genome.

Protein NP_853190.1 was a completely unassigned protein. Our method predicted peptidase_M23 (Peptidase family M23) domain relationship in the protein. Members of this family are zinc metallopeptidases and have a characteristic HxH motif [36], and the current gene product also preserved this functional motif in the unassigned region. We found this domain in *Mycoplasma gallisepticum* only through indirect searches, and the unassigned sequence has less than 20% sequence identity with the typical peptidase_M23 members, albeit with few indels in the alignment (Figure 1). Perhaps, the low sequence identity could explain why this is not associated with domain in the direct searches. Peptidase_M23 domain is present in only two other *Mycoplasma* members (*Mycoplasma mobile *and *Mycoplasma pulmonis)*. Interestingly, chaperonin (cpn60 or GroEL) domain is absent from these species but is present in *Mycoplasma gallisepticum *genome. Peptidases and chaperonins are components of protein homeostatic mechanisms. Molecular chaperones promote protein folding and prevent protein misfolding and aggregation, while certain proteases function primarily to degrade improperly folded proteins [37, 38]. It has been hypothesized that the protein homeostatic process in Mollicute organisms has shifted through evolution towards favoring protein degradation rather than protein folding [39]. Since peptidase_M23 is present only in *M. mobile *and *M. pulmonis *(Figure 2) along with other peptidases where GroEL is completely absent from the genomes, this may explain the need for higher peptidases to degrade improperly folded proteins. Whereas, in *M. gallisepticum, *the presence of GroEL reduces the pressure on peptidases like peptidase_M23 and sequences could have diverged substantially.

The full-length region of the sequence ID NP_852865.1 was unassigned; that is, no sequence domains were observed and recorded. Our method indirectly assigned amino terminal Lactamase_B and carboxy terminal RMMBL (RNA-metabolizing metallo-beta-lactamase) domains in the sequence. In the initial PSI-BLAST search against nonredundant database, it has picked up which belongs to more than 100 different species, including *Homo sapiens, *at very low expectation values. In the Hmmpfam search, all the hits showed identical domain architectures in all the sequences with amino terminal Lactamase_B and carboxy terminal RMMBL domains and with very good *E* values. The metallo-beta-lactamase fold contains five sequence motifs. The first four motifs are found in Lactamase_B (PF00753) and are common to all metallo-beta-lactamases. The fifth motif appears to be specific to function. RMMBL represents the fifth motif from metallo-beta-lactamases involved in RNA metabolism.

**Figure 3:**
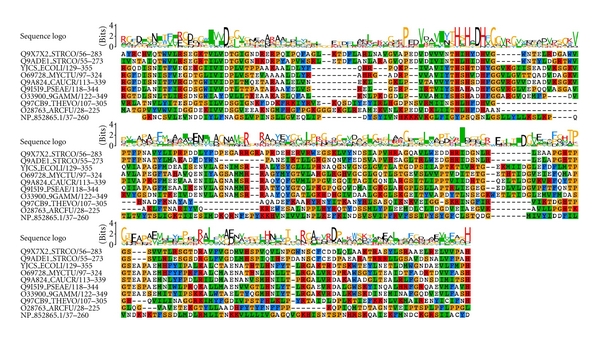
Multiple sequence alignment between Lactamase_b family representative sequences and unassigned protein sequence (NP_852865.1) from *M. gallisepticum *genome. Lactamase_b sequences obtained from Pfam database.

**Figure 4:**
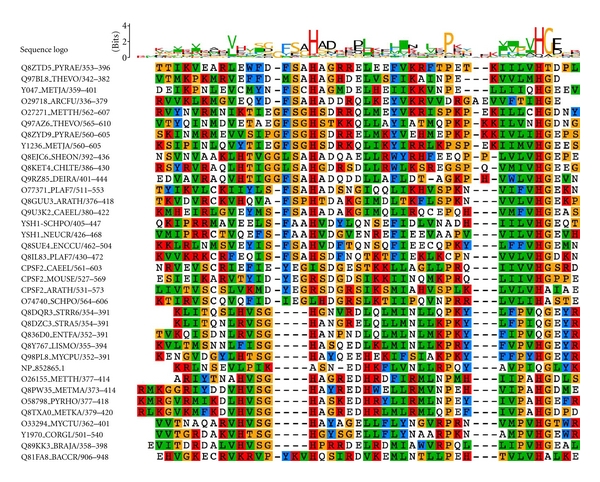
Multiple sequence alignment between RMMBL family representative sequences and unassigned protein sequence (NP_852865.1) from *M. gallisepticum *genome. RMMBL sequences obtained from Pfam database.

**Figure 5:**
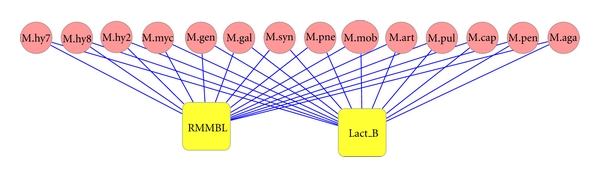
Presence of RMMBL and Lactamase_B domains and domain architecture in different *Mycoplasma* genomes. Domains are in square box and species in the circles. Edges represent presence of that domain in that species.

Multiple sequence alignment of predicted regions with typical Lactamase_B and RMMBL (Figures 3 and 4) revealed that the most residues that are typical to the family are not conserved. The domains and domain architecture is conserved across Mycoplasmataceae members (Figure 5). It has been documented that presence of paralogs in *Mycoplasma genitalium* (MG139 and MG423) and *Mycoplasma pneumoniae* (MPN280 and MPN261) along with other bacteria [40, 41] could be as inactive forms. These inactive forms could be confined to modularity function helping in regulating enzymatic activity as already suggested by Aravind [41]. Acquisition of new functions beyond the ancestral enzymatic one is also possible [41]. Due to low sequence identities (<20%) with typical Lactamase_B members, in *Mycoplasma gallisepticum *initially there was only one copy of Lactamase_B domain in the genome (NP_852802.1). Our analysis revealed that there is a putative paralog of this domain in this genome, like other *Mycoplasma* genomes.

**Figure 6:**
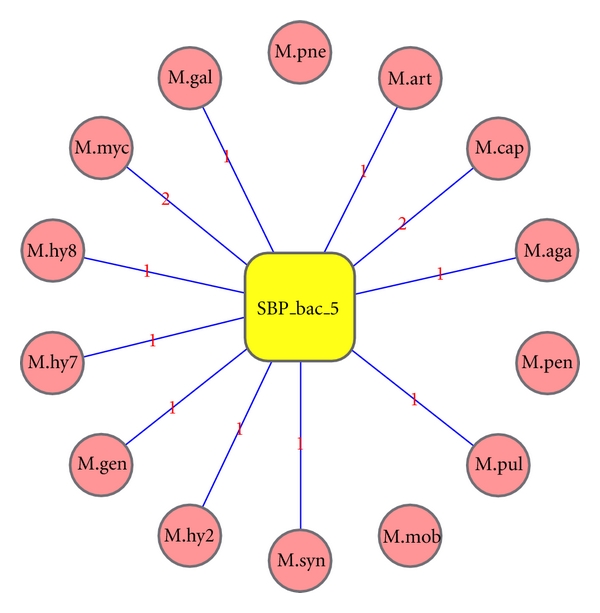
Comparison of SBP_bac_5 domain across different *Mycoplasma* genomes. SBP_bac_5 domain present in all the *Mycoplasma* genomes but in *Mycoplasma mobile*, *Mycoplasma pneumoniae*, and *Mycoplasma synoviae*. All the genomes have single copy of SBP_bac_5 domain except *Mycoplasma mycoides* and *Mycoplasma capricolum* where two copies were found in the genome.

SBP_bac_5 (bacterial extracellular solute-binding proteins, family 5) domain relationship is established in NP_853298.1 (see Table 2), which was initially full-length unassigned sequence. Cross-genome comparisons revealed that this domain is present in all the *Mycoplasma* species, except *Mycoplasma mobile*, *Mycoplasma pneumoniae*, and *Mycoplasma synoviae* (Figure 6). This domain is involved in peptide and nickel transportation. Mycoplasmas have reduced genome size and are highly dependent on the environment for nutrient abortion [35]. The presence of extra SBP_bac_5 domain could help in the peptide uptake by the organisms.

*Mycoplasma* species were classified into six different groups according to host specificities (as mentioned earlier), and the newly predicted domains were classified based on the host specificities (Table 3; see Supplementary Table S1 in Supplementary Material available online at doi: 10.1155/2011/878973). There were few domains, which are group specific, while the majority are found in all the groups. The group specific domains perhaps imply their selectivity in the hosts owing to function which may be directly or indirectly required for its survival. We found that the two domains namely, HNH (endonuclease) and HTH_5 (helix turn helix motif containing transcription factor), are specific to *M. mobile* (found in fresh water Tench fish—*Tinca tinca*). Five domains namely, GMP_synt_C (GMP synthase CTD), HHH (helix-hairpin-helix motif involved in DNA binding), Methyltransf_3 (O-methyltransferases), SBP_bac_1 (Bacterial extracellular solute-binding protein), and Transposase_mut (Transposase, Mutator family with DNA-based transposition activity), were found to be primarily in human-specific and primate group-specific pathogens. Most of these species-specific domains are involved in DNA binding and have transcription factor functions. One of them, GMP_synt_C (GMP synthase CTD), is associated with GATase (Glutamine amidotransferase class-I) and Peptidase_C26 domains to form a gene product in *M. penetrans* involved in GMP biosynthesis. Amongst the human- and primate-specific pathogens, *M. penetrans* has the largest genome (1,358,633 nt) and maximum number of proteins (1037) among all 14 *Mycoplasma* species analyzed in this study (Table 1), suggesting that this organism may possess additional genetic information involved in its unique infection process. This organism lacks pyrimidine biosynthetic machinery but using orotate-related metabolism (again unique to *M. penetrans*) circumvents this problem [33]. On the other hand, presence of purine biosynthesis (GMP synthase) related protein assists on the purine part of nucleotide biosynthesis. Also, the larger size of genome and number of proteins present underlines presence of GMP_synt_C domain specific to *M. penetrans*. Such an inspection of domain architectures in proteins containing these newly predicted domains was carried out for all host-group specific domains. It revealed that, except for GMP_synt_C, all other domains are present as single domains in complete protein sequences. Most of the newly predicted domains are transcription factors not only involved in nucleotide biosynthesis but also specifically involved in the regulation of solute transport. This fact emphasizes the importance of solute transfer across the membrane in conditions of minimal genomes. Host-group-wise comparative analysis revealed that the TGS domain is present in two groups, rodents and ovine/caprine. Even within rodent-specific pathogens, it is present in only *M. arthritidis_158L3_1*, whereas; it is present in all three species of the ovine/caprine host group. TGS domain is named after threonyl-tRNA synthetase (ThrRS), GTPase, and guanosine-3′,5′-bis(diphosphate) 3′-pyrophosphohydrolase (SpoT). Its presence in proteins like GTPases suggests its role in ligand (nucleotide) binding or some regulatory function, but it has no direct information about function [35]. However, in *M. mycoides*, it is present in association with other domains in two different proteins. One of them is GTP diphosphokinase involved in guanosine tetraphosphate metabolic process explaining the possible involvement of TGS domain in nucleotide biosynthetic machinery. Here, *M. mycoides,* which also infects cattle (causing contagious bovine pleuropneumonia (CBPP)), has the second largest genome (1,211,703 nt) and number of proteins (1016) in the 14 *Mycoplasma* species under consideration (Table 1), explaining the presence of additional genetic information [34].

Some of the domains are specific to motile group, for example, HHH, HNH, HTH_5, Peptidase_M23, and SBP_bac_1 are specific to motile group, whereas GMP_synt_C, Methyltransf_3, NusB, TGS, and Transposase_mut domains are specific to nonmotile group (Supplementary Table S2). Inspecting the domain architectures for all the domains specific to the motility group, we found that they were not associated with any other domain in the complete protein sequence, except for the HHH domain in *M. pneumoniae*. Even in *M. pneumonia*, HHH (helix-hairpin-helix motif—small DNA-binding motif) was associated with three different ligase domains involved in replication, repair, and recombination events. Therefore, although there is no obvious link between the presence and absence of these domains and motility function, these distant relationships perhaps acquired new function, which may be required for motility of the pathogens.

## 4. Conclusions

The investigation in the sequence information among closely related genomes helps in tracing of appearance, disappearance, and reappearance of genes or their close homologues in closely related bacterial genomes. Generally, functional annotation transfer is accomplished by phylogenomics-based methods that exploit strong phylogenetic relationship and based on the closest orthologue identified [42]. Apart from different sequence homology-based methods, microarray expression data along with machine learning techniques like Support Vector Machines (SVM) are integrated together for functional annotations [43]. Although use of such methods will be useful, GO annotations could be more comprehensive with regards to the biological process part or the cellular component part than for the exact molecular function [44]. Protein classification methods along with gene ontology terms are very useful tools in protein functional annotation. However, the best hit with respect to sequence identity may not be the correct protein to be used for annotation transfer since paralogous protein sequences from the same organism do share high identity but function may vary. 

In this study, newly and indirectly identified domains in *Mycoplasma gallisepticum* have been compared across 14 *Mycoplasma* species. This study showed that some of the newly identified domains are specific to *Mycoplasma gallisepticum* genome. Such genome-specific domains will perhaps provide important clues to the physiological and pathogenic specificities of the genome.

## Supplementary Material

Supplementary Table S1: Host-group wise *Mycoplasma* species and the presence of PURE predicted domains in these groups. 6 groups for 14 species represent here the domains specific to a single group. Accordingly, 7 domains were found to be group specific. 5 domains (highlighted in yellow) were specific to Human/Primate group whereas 2 domains (highlighted in blue) were specific to Fish group.
Supplementary Table S2: Grouping of *Mycoplasma* on the basis of motility with representation of PURE predicted domains present in these groups. 5 species are motile while 9 are non-motile representing group-wise counts for domains common to all members of a group whereas domains specific to a group are also reported. Each group reported 5 domains specific to it. They are highlighted accordingly (yellow for motile and blue for non-motile). Presence of a domain is indicated by ‘1' and absence by ‘0'.

## Figures and Tables

**Table 1:** 14 *Mycoplasma* species considered in this study. Host-group specificity and motility information is provided with genome size and total number of proteins present in the individual species.

Organism	Genome size (nt)	No. of proteins	Host-group specificity	Motility
*M. agalactiae_PG2*	877,438	742	Ovine/caprine	Nonmotile
*M. arthritidis_158L3_1*	820,453	631	Rodents	Nonmotile
*M. capricolum_ATCC_27343*	1,010,023	812	Ovine/caprine	Nonmotile
*M. gallisepticum *	1,012,800	726	Avian	Motile
*M. genitalium*	580,076	477	Human/primates	Motile
*M. hyopneumoniae_232*	892,758	691	Swine	Nonmotile
*M. hyopneumoniae_7448*	920,079	657	Swine	Nonmotile
*M. hyopneumoniae *−8	897,405	657	Swine	Nonmotile
*M. mobile *	777,079	633	Fish	Motile
*M. mycoides*	1,211,703	1016	Ovine/caprine	Nonmotile
*M. penetrans*	1,358,633	1037	Human/primates	Nonmotile
*M. pneumoniae*	816,394	689	Human/primates	Motile
*M. pulmonis*	963,879	782	Human/primates and Rodents	Motile
*M. synoviae_53*	799,476	659	Avian	Nonmotile

**Table 2:** New domain architectures of 42 *Mycoplasma gallisepticum* proteins. For each protein reference ID is given in column two along with protein length. Fully associated domains are indicated with blue color, partially associated domains with brown color, and yellow color indicated the domains already associated with protein. Each domain is indicated by its name, starting and ending residues. In column four D represents Domain, Da indicates domain architectures. If particular domain or domain architecture present in the proteome it is indicated by P indicates present and domain/domain architecture not present in the existing proteome is indicated by NP meaning not present. Symbol * indicates that; this protein sequence is completely unassigned before out method, ^@^ indicated unique domain/domain architecture and ^&^ indicated average sequence identities.

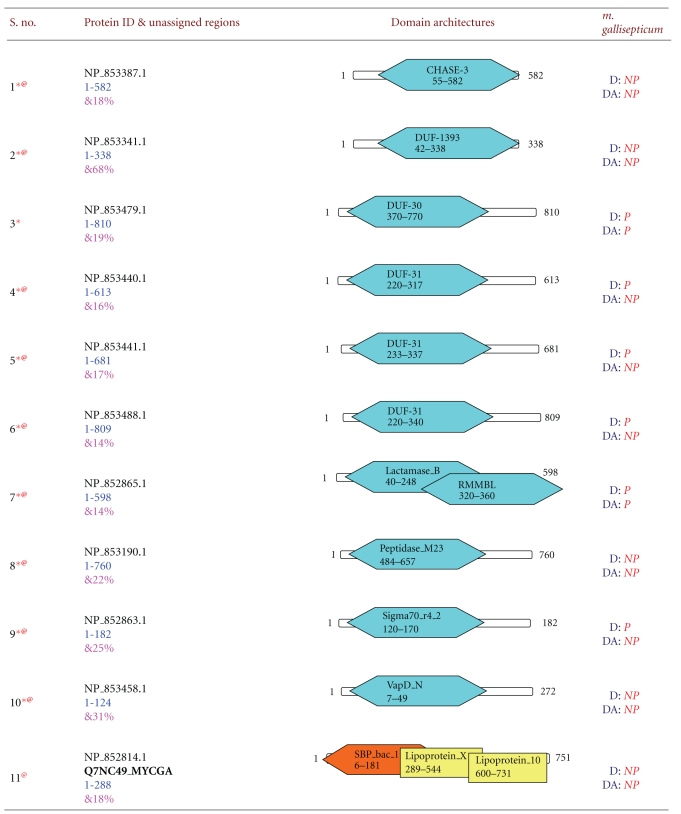	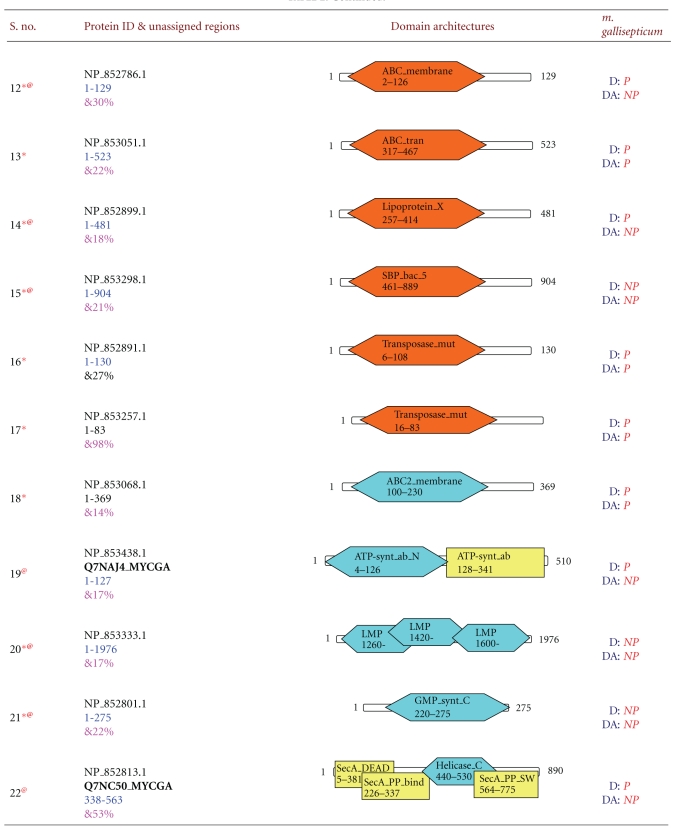	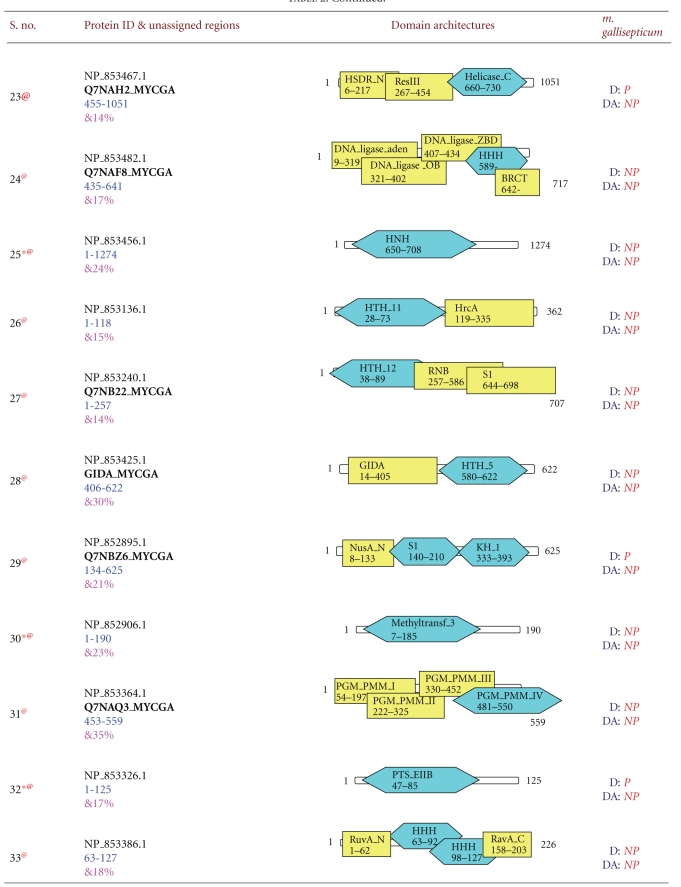	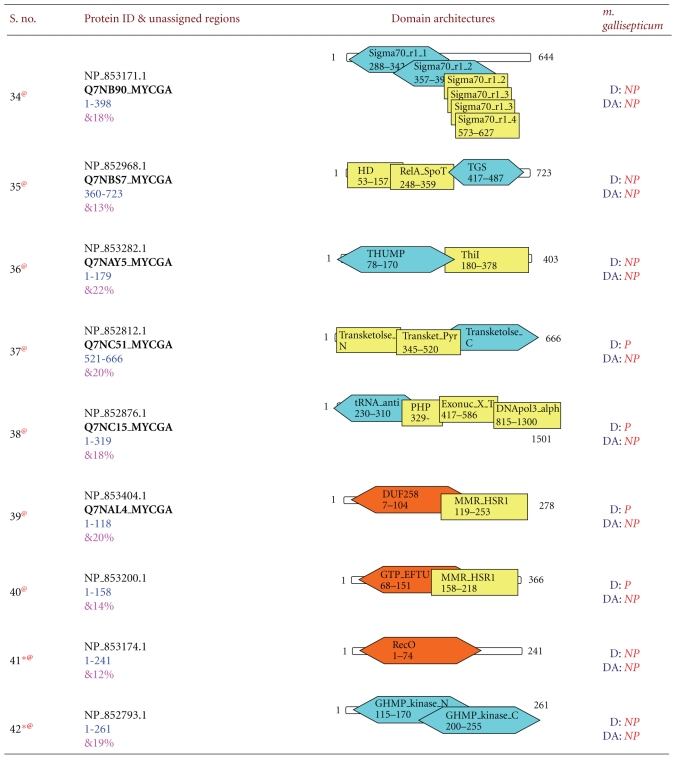

**Table 3:** Comparison of PURE-predicted domains *in M. gallisepticum* to the Pfam-reported domains in the 14 *Mycoplasma* species. Species grouped based on host specificities of the individual *Mycoplasma* species.

Host group	Human (+primates)	Avian	Swine	Rodents	Ovine/caprine	Fish
No. of *Mycoplasma* spp.	4	2	3	2	3	1
Species	*M. genitalium*	*M. synoviae_53*	*M. hyopneumoniae_232*	*M. arthritidis_158L3_1*	*M. agalactiae_PG2*	*M. mobile*
	*M. penetrans*	*M. gallisepticum*	*M. hyopneumoniae_7448*	*M. pulmonis*	*M. capricolum_ATCC_27343*	
	*M. pneumoniae*		*M. hyopneumoniae-8*		*M. mycoides*	
	*M. pulmonis*					
No. of domains	(25+26+24+27) 36	(23+24) 27	(24+24+24) 24	(24+27) 29	(25+24+27) 29	25
No. of intragroup common domains	19	20	24	22	21	25
No. of group-specific domains	5	0	0	0	0	2
