# Supplementary material for: Cross-Genome Comparisons of Newly Identified Domains in Mycoplasma gallisepticum and Domain Architectures with Other Mycoplasma species
Source: Comp Funct Genomics. 2011 Aug 8;2011:878973. doi: 10.1155/2011/878973 (PMC3155973; doi:10.1155/2011/878973)
Supplement: Supplementary file 1 — Supplementary Table S1: Host-group wise Mycoplasma species and the presence of PURE predicted domains in these groups. 6 groups for 14 species represent here the domains specific to a single group. Accordingly, 7 domains were found to be group specific. 5 domains (highlighted in yellow) were specific to Human/Primate group whereas 2 domains (highlighted in blue) were specific to Fish group. Supplementary Table S2: Grouping of Mycoplasma on the basis of motility with representation of PURE predicted domains present in these groups. 5 species are motile while 9 are non-motile representing group-wise counts for domains common to all members of a group whereas domains specific to a group are also reported. Each group reported 5 domains specific to it. They are highlighted accordingly (yellow for motile and blue for non-motile). Presence of a domain is indicated by ‘1' and absence by ‘0'. [file 878973.f1.pdf]

Supplementary Table S1: Host-group wise *Mycoplasma* species and the presence of PURE predicted domains in these groups. 6 groups for 14 species represent here the domains specific to a single group. Accordingly, 7 domains were found to be group specific. 5 domains (highlighted in yellow) were specific to Human/Primate group whereas 2 domains (highlighted in blue) were specific to Fish group.

DOMAINS	HUMAN/ PRIMATE	AVIAN	SWINE	RODENTS	OVINE/ CAPRINE	FISH	Domainwise Count
AAA	1	1	1	1	1	1	6
Anticodon_1	1	1	1	1	1	1	6
ATP-synt_ab_C	1	1	1	1	1	1	6
ATP-synt_ab_N	1	1	1	1	1	1	6
BPD_transp_1	1	1	1	1	1	1	6
CHASE3	0	0	0	0	0	0	0
DUF1393	0	0	0	0	0	0	0
DUF30	0	0	0	0	0	0	0
DUF31	0	0	0	0	0	0	0
LMP	0	0	0	0	0	0	0
Ferritin	1	1	0	1	0	0	3
GMP_synt_C	1	0	0	0	0	0	1
Helicase_C	1	1	1	1	1	1	6
HGTP_anticodon	1	1	1	1	1	1	6
HHH	1	0	0	0	0	0	1
HNH	0	0	0	0	0	1	1
HTH_11	1	0	0	0	1	1	3
HTH_12	0	0	0	0	0	0	0
HTH_5	0	0	0	0	0	1	1
KH_1	1	1	0	0	1	0	3
Lactamase_B	1	1	1	1	1	1	6
RMMBL	1	1	1	1	1	1	6
MatE	1	1	1	1	0	0	4
Methyltransf_3	1	0	0	0	0	0	1
MFS_1	1	1	1	1	1	1	6
NusB	1	1	1	1	1	0	5
Peptidase_M23	1	0	0	1	0	1	3
PGM_PMM_IV	1	0	0	0	1	1	3
PTS_EIIB	1	1	1	1	1	1	6
SBP_bac_1	1	0	0	0	0	0	1
Sigma70_r1_1	0	0	0	0	0	0	0
Sigma70_r1_2	1	1	1	1	1	1	6
Sigma70_r4_2	1	1	0	1	1	1	5
TGS	0	0	0	1	1	0	2
Transketolase_C	1	1	1	1	1	1	6
tRNA_anti	1	1	1	1	1	1	6
Trypan_PARP	0	0	0	0	0	0	0
Vps55	0	0	0	0	0	0	0
VapD	0	0	0	0	0	0	0
Lipoprotein_10	1	1	1	1	1	0	5

Lipoprotein_X	1	1	1	1	1	0	5
DEAD	1	1	1	1	1	1	6
ABC_membrane	1	1	1	1	1	1	6
ABC_tran	1	1	1	1	1	1	6
DUF258	1	1	1	1	1	1	6
GTP_EFTU	1	1	1	1	1	1	6
RecO	1	0	0	1	1	0	3
SBP_bac_5	1	1	1	1	1	0	5
Transposase_mut	1	0	0	0	0	0	1
Groupwise Counts	36	27	24	29	29	25	170

Supplementary Table S2: Grouping of Mycoplasma on the basis of motility with representation of PURE predicted domains present in these groups. 5 species are motile while 9 are non-motile representing group-wise counts for domains common to all members of a group whereas domains specific to a group are also reported. Each group reported 5 domains specific to it. They are highlighted accordingly (yellow for motile and blue for non-motile). Presence of a domain is indicated by ‘1’ and absence by ‘0’.

GROUP	MOTILE	NON-MOTILE
No. of *Mycoplasma* species	5	9
Species	*M. gallisepticum*	*M. agalactiae_PG2*
	M. genitalium	*M. arthritidis_158L3_1*
	M. mobile	*M. capricolum_ATCC_27343*
	M. pneumoniae	*M. hyponeumoniae_232*
	M. pulmonis	*M. hyponeumoniae_7448*
		M. hyponeumoniae-8
		M. mycoides
		M. penetrans
		M. synoviae_53
No. of domains	(23+25+25+24+27) 34	(25+24+24+24+24+24+27+26+24) 34
No. of intra-group common domains	17	16
No. of group specific domains	5	5
**DOMAINS**	**MOTILE**	**NON-MOTILE**
AAA	1	1
Anticodon_1	1	1
ATP-synt_ab_C	1	1
ATP-synt_ab_N	1	1
BPD_transp_1	1	1
CHASE3	0	0
DUF1393	0	0
DUF30	0	0
DUF31	0	0
LMP	0	0
Ferritin	1	1
GMP_synt_C	0	1
Helicase_C	1	1
HGTP_anticodon	1	1
HHH	1	0
HNH	1	0
HTH_11	1	1
HTH_12	0	0

HTH_5	1	0
KH_1	1	1
Lactamase_B	1	1
RMMBL	1	1
MatE	1	1
Methyltransf_3	0	1
MFS_1	1	1
NusB	0	1
Peptidase_M23	1	0
PGM_PMM_IV	1	1
PTS_EIIB	1	1
SBP_bac_1	1	0
Sigma70_r1_1	0	0
Sigma70_r1_2	1	1
Sigma70_r4_2	1	1
TGS	0	1
Transketolase_C	1	1
tRNA_anti	1	1
Trypan_PARP	0	0
Vps55	0	0
VapD	0	0
Lipoprotein_10	1	1
Lipoprotein_X	1	1
DEAD	1	1
ABC_membrane	1	1
ABC_tran	1	1
DUF258	1	1
GTP_EFTU	1	1
RecO	1	1
SBP_bac_5	1	1
Transposase_mut	0	1
GroupWise Counts	34	34
